# Environmental enrichment is associated with changes in the gut microbiome and resistome of captive pythons

**DOI:** 10.1371/journal.pone.0360735

**Published:** 2026-11-04

**Authors:** Ji Chen, Yu-Qi Cheng, Lin-Ping Fan, Xia Zhou, Jiu-Yan Jiang, Fei Wu, Ping-Wei Liang, Yang Liu, Zhi-Li Zuo, Guang-Xiang Zhu

**Affiliations:** 1 College of Life Science, Sichuan Agricultural University, Yaan, P. R. China; 2 Chengdu Zoo, Chengdu, P. R. China; Anhui University, CHINA

## Abstract

Environmental enrichment is widely used to improve the welfare of captive animals, yet its effects on reptilian gut microbiota remain poorly understood. Here, we used shotgun metagenomic sequencing to investigate the gut microbiome and resistome of two captive python species: Burmese python (*Python bivittatus)* and reticulated python (*Malayopython reticulatus)*, before and after environmental enrichment. We found that environmental enrichment was associated with increased α-diversity in Burmese pythons, as indicated by significantly higher ACE and observed species indices, while NMDS ordination revealed differences in the observed patterns of community composition between the two species. Pseudomonadota and Bacteroidota were dominant phyla in the gut microbiota of both python species, but their relative abundances responded differently to enrichment between hosts. Functional analyses suggested an enhanced metabolic potential in Burmese pythons, particularly in carbohydrate metabolism and environmental information processing, accompanied by increased abundance of carbohydrate-active enzymes. In addition, enrichment may influence the gut resistome, with Pseudomonadota contributing the majority of antibiotic resistance genes. Overall, our results demonstrate that environmental enrichment measures, such as dietary changes and improvements in water and air quality, are associated with changes in the gut microbiome and resistome of captive pythons in a host-dependent manner, highlighting the combined roles of diet and environmental conditions in the observed changes in the gut microbiota. These findings provide exploratory insights into the functional responses and host–microbiome evolution of reptile gut microbiota to captivity and have important implications for the management and welfare of captive animals.

## Introduction

Captive breeding has been widely used as an important strategy for wildlife conservation, supporting species protection, scientific research, and public education [1,2]. However, captive conditions can alter animal behavior, diet, and physiology, potentially reducing adaptability and increasing health risks [3,4]. Therefore, improving husbandry practices while maintaining animal welfare remains a major challenge in captive management.

Environmental enrichment is a key approach to enhance animal welfare by increasing environmental complexity and optimizing living conditions [5]. Growing evidence suggests that enrichment can also influence gut microbiota and immune parameters [6–8]. For example, studies in mammals have shown that enrichment can modulate microbial composition and promote the production of beneficial metabolites such as short-chain fatty acids [9,10]. Similar effects have been observed in fish, where enrichment enhances microbial diversity and improves survival [11].

Despite these advances, the effects of environmental enrichment on reptilian gut microbiota remain poorly understood, particularly in snakes. As ectothermic vertebrates with distinct feeding strategies and physiological traits, snakes may exhibit unique microbiome responses to environmental changes. Understanding these responses is essential for improving captive management and conservation outcomes.

The Burmese python (*Python bivittatus*) and reticulated python (*Malayopython reticulatus*) are two ecologically distinct snake species with differences in body size, habitat, and feeding behavior, which may lead to host-specific microbiome responses. In this study, we used shotgun metagenomic sequencing to compare gut microbial diversity, potential functions, and resistome profiles in both species before and after environmental enrichment.

We hypothesized that environmental enrichment would be associated with (1) shifts in gut microbial diversity and community composition in captive pythons and (2) alterations in antibiotic resistance gene (ARG) profiles. Separately, we addressed the following exploratory research question: How do the observed gut microbiome changes following environmental enrichment compare between Burmese and reticulated pythons? Our findings provide new insights into the role of husbandry practices and offer a microbial perspective for improving animal welfare and conservation management.

## Materials and methods

### Ethics statement

The animal use protocol listed has been reviewed and approved by the Sichuan Agricultural University Animal Ethics and Welfare Committee (Approval

No. 20240261, approved on 28 February 2024).

### Sample collection

Fresh fecal samples were collected from captive Burmese pythons (*Python bivittatus*) and reticulated pythons (*Malayopython reticulatus*) in April–May 2024 2024 (before enrichment) and October–November 2024 (after enrichment) using sterile cotton swabs (S1 Table). Each swab was immediately placed into RNase-free centrifuge tubes and stored for subsequent DNA extraction. For Burmese pythons, six samples were collected before enrichment and six samples after enrichment (BP-B, n = 6; BP-A, n = 6). For reticulated pythons, three samples were collected before enrichment and three samples after enrichment (RP-B, n = 3; RP-A, n = 3). Individual identification numbers were retained across sampling periods to ensure that pre- and post-enrichment samples from the same animal were paired for subsequent analyses. Both python species were housed in adjacent exhibition enclosures under comparable management conditions. Environmental enrichment was implemented in June 2024. The enrichment measures included: (1) dietary changes: both pythons species were fed white mice before the enrichment and rabbits afterward, and their food intake increased. The snakes were maintained according to the routine husbandry and feeding practices of Chengdu Zoo. Although routine feeding records were available, the interval between the last feeding event and fecal sample collection was not systematically recorded for each individual and was therefore not included as a covariate in the statistical analyses. (2) the addition of a flowing water source with a filtration system to mimic a natural pond and improve water quality; (3) the installation of an air purification system to enhance air circulation and quality; (4) the installation of a misting system to increase ambient humidity; (5) the installation of a lighting system with UVA light to regulate circadian rhythms and meet basking requirements [12]; (6) the addition of artificial rocks and perches to increase environmental complexity and promote climbing and exploratory behavior; and (7) the addition of natural elements such as plants and soil to simulate the natural habitat. Fecal samples were collected non-invasively without direct animal handling or disturbance.

### Total DNA extraction and library construction

Total genomic DNA was extracted from fecal samples using the Tian-gen DNA Stool Mini Kit (TIANGEN Biotech, Beijing, China) according to the manufacturer’s instructions with minor modifications. DNA concentration and purity were measured using a NanoDrop 3300 (Thermo Scientific, Shanghai, China). Sequencing was performed by Novogene (Beijing, China). Approximately 1 μg of genomic DNA from each sample was randomly sheared into fragments of approximately 350 bp using a Covaris ultrasonic homogenizer for library construction. Library preparation included end repair, A-tailing, sequencing adapter ligation, purification, and PCR amplification. Fragment integrity and insert size were assessed using a Bioanalyzer system. Libraries were quantified by qPCR (effective library concentration >3 nM) after confirming the expected parameters. After quality control, libraries were pooled according to the required concentration and target sequencing depth and subjected to paired-end sequencing (PE150).

### Data processing

Raw sequencing data were first subjected to quality control using fastp (v0.24.0) [13]. The clean reads were then aligned to the host genome using Bowtie2 (v2.5.4) to remove host-derived sequences [14]. The resulting clean reads were assembled using MEGAHIT (v1.2.0) [15]. The assembled scaffolds were split at N positions to generate N-free scaffolds. Open reading frames (ORFs) were predicted using MetaGeneMark (v3.38), and a non-redundant gene catalog was constructed using CD-HIT (v4.8.1).

### Species annotation

Clean reads from each sample were aligned to the non-redundant gene catalog using Bowtie2 (v2.5.4) to calculate gene abundance. Genes with low abundance (reads ≤ 2) were filtered out to generate the final gene catalog (unigenes) for subsequent analysis. Gene abundance in each sample was calculated based on the number of aligned reads and gene length. Unigenes were annotated by aligning sequences against the microNR database using DIAMOND (v2.1.10) [16]. Taxonomic assignment was performed based on the lowest common ancestor (LCA) algorithm, and gene abundance across taxonomic categories was summarized accordingly [17].

### Diversity analysis

Abundance tables at different taxonomic levels were used to generate clustering heatmaps and Venn diagrams of unique genes. Alpha-diversity indices (Shannon, Simpson, ACE and observed species) were calculated based on genus-level abundance profiles. For comparisons between the pre- and post-enrichment conditions within the same individuals, the Wilcoxon signed-rank test was used because the observations were paired. Each pre-enrichment sample was matched to the corresponding post-enrichment sample from the same snake based on individual identification. Beta-diversity was evaluated using non-metric multidimensional scaling (NMDS) analysis based on Bray–Curtis distance using the ade4 package in R (v4.5.0). Differential abundance analysis was performed using the Metastats method, and *p* values were adjusted using the Benjamini–Hochberg false discovery rate (FDR) procedure; taxa with an FDR-adjusted *p* value < 0.05 were considered statistically significant. Differentially abundant taxa were identified using linear discriminant analysis effect size (LEfSe) with an LDA score threshold of 3 [18,19].

### Functional annotation

Unigenes were aligned against the KEGG and CAZy databases for functional annotation using DIAMOND (v2.1.10). For each sequence, the best hit was selected for subsequent analysis. The relative abundance of functional categories at different levels was calculated based on gene abundance [20,21].

### Resistance gene annotation

Unigenes were aligned against the Comprehensive Antibiotic Resistance Database (CARD) using the Resistance Gene Identifier (RGI) software (v6.0.3). Based on the RGI results and unigene abundance data, the relative abundance of each antibiotic resistance ontology (ARO) term was calculated. The relative abundance of AROs was then used to generate chord diagrams and species annotation analyses to visualize the distribution of antibiotic resistance genes [22].

## Results

### Sequencing information

This study obtained gut metagenomic sequencing data from 18 samples (S2 Table). The raw base output ranged from 6.28 Gb to 8.08 Gb, with an average of 7.19 Gb. The clean data ranged from 6.18 Gb to 8.02 Gb, with an average of 7.10 Gb.

The proportion of high-quality bases (Q20) ranged from 97.52% to 97.89%, while bases with Q30 ranged from 92.72% to 93.76%. The GC content across samples ranged from 40.60% to 45.45%, with an average of 41.98%. The sequencing efficiency ranged from 98.36% to 99.28%.

The Venn diagram showed that 1,935,666 genes were shared among the four groups. The number of unique genes in BP-B, RP-B, BP-A, and RP-A was 20,518, 18,290, 46,242, and 18,990, respectively. Overall, the BP-A and RP-A groups exhibited higher gene numbers than their respective pre-enrichment groups (Fig 1).

**Fig 1:**
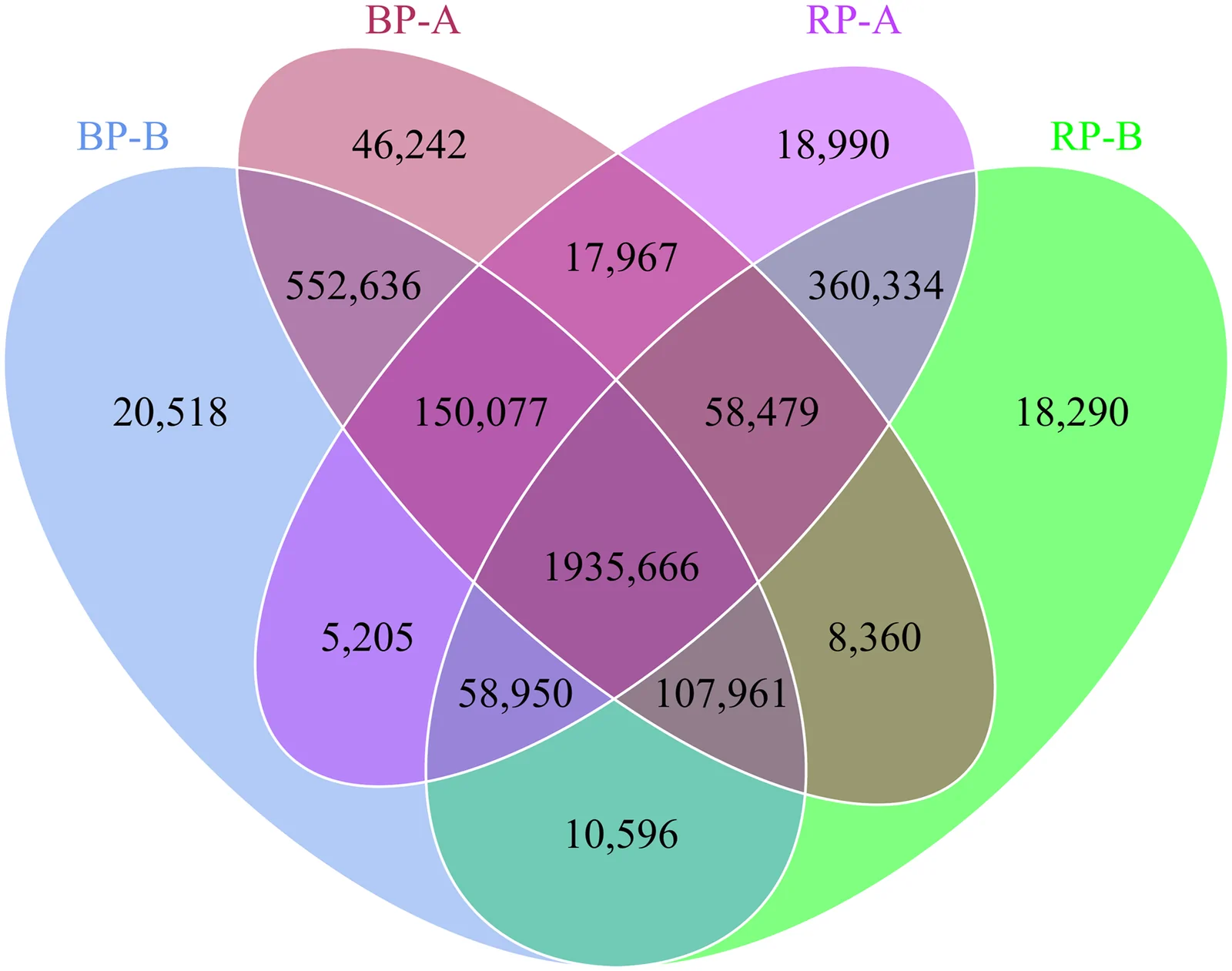
Venn diagram showing unique genes among the four groups.

### Diversity and composition of the gut microbiota

Based on genus-level relative abundance profiles of the two python species, alpha diversity analysis was performed. Significant differences were observed in ACE (*p* = 0.031) and observed species (*p* = 0.036) indices between BP-B and BP-A, while no significant differences were detected for the other indices (Fig 2A, S3 Table).

**Fig 2:**
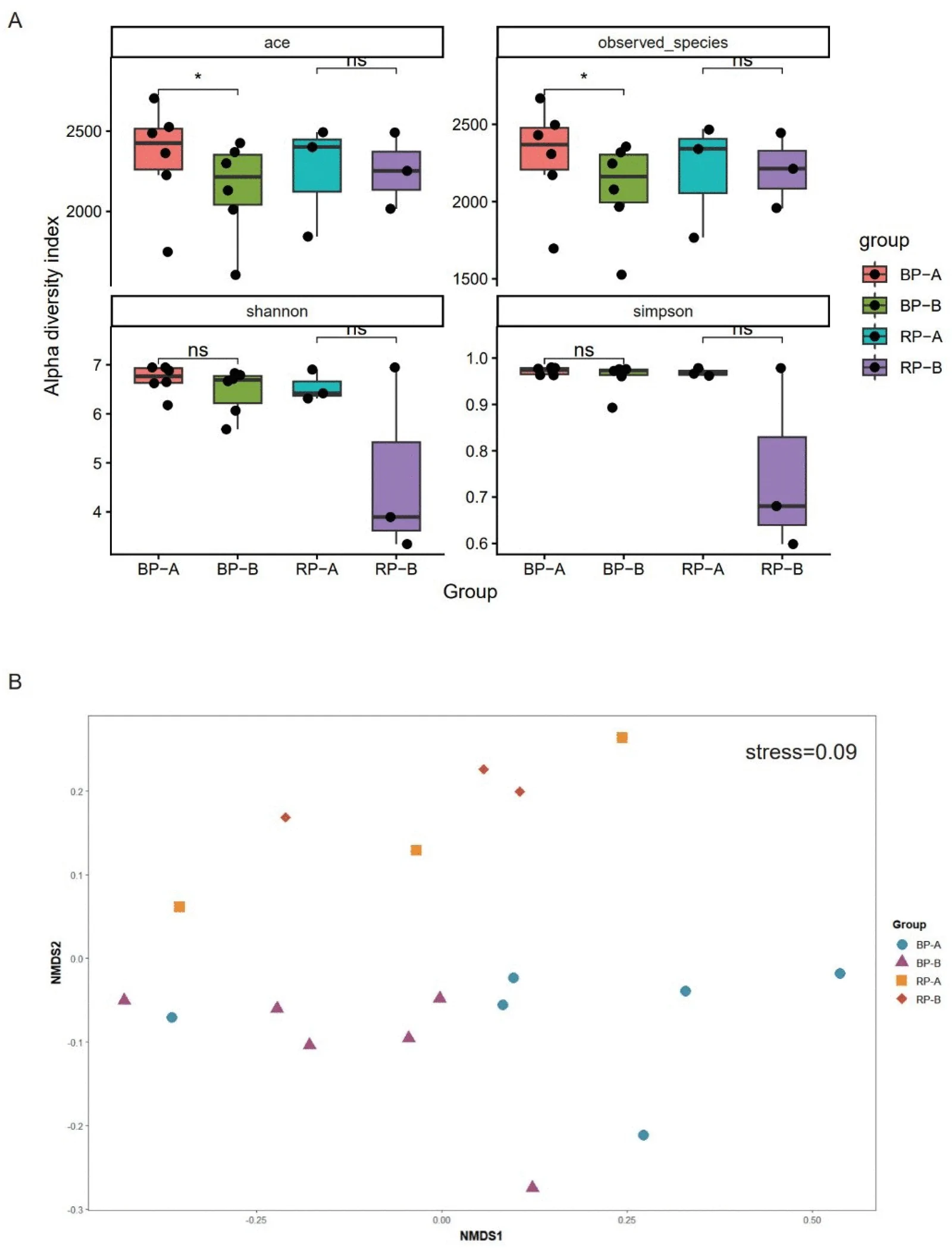
Gut microbial diversity in two python species before and after environmental enrichment. (A) Alpha-diversity indices (ACE, observed species, Shannon, and Simpson). Pre- and post-enrichment samples from the same individuals were compared within each species using the Wilcoxon signed-rank test (* *p* < 0.05; ** *p* < 0.01). (B) Non-metric multidimensional scaling (NMDS) ordination showing sample distribution patterns based on Bray–Curtis dissimilarities calculated from genus-level relative abundance profiles (stress = 0.09).

In the NMDS ordination, samples from the same species tended to cluster together, suggesting species-associated patterns in community composition. The low stress value (0.09) indicated a good representation of the community structure (Fig 2B).

At the phylum level, the top five phyla by relative abundance were Pseudomonadota (8.88% ± 2.37%), Bacteroidota (2.83% ± 1.28%), Bacillota (1.20% ± 0.18%), Actinomycetota (0.72% ± 0.08%), and Campylobacterota (0.51% ± 0.15%) (Fig 3A, S4 Table).

**Fig 3:**
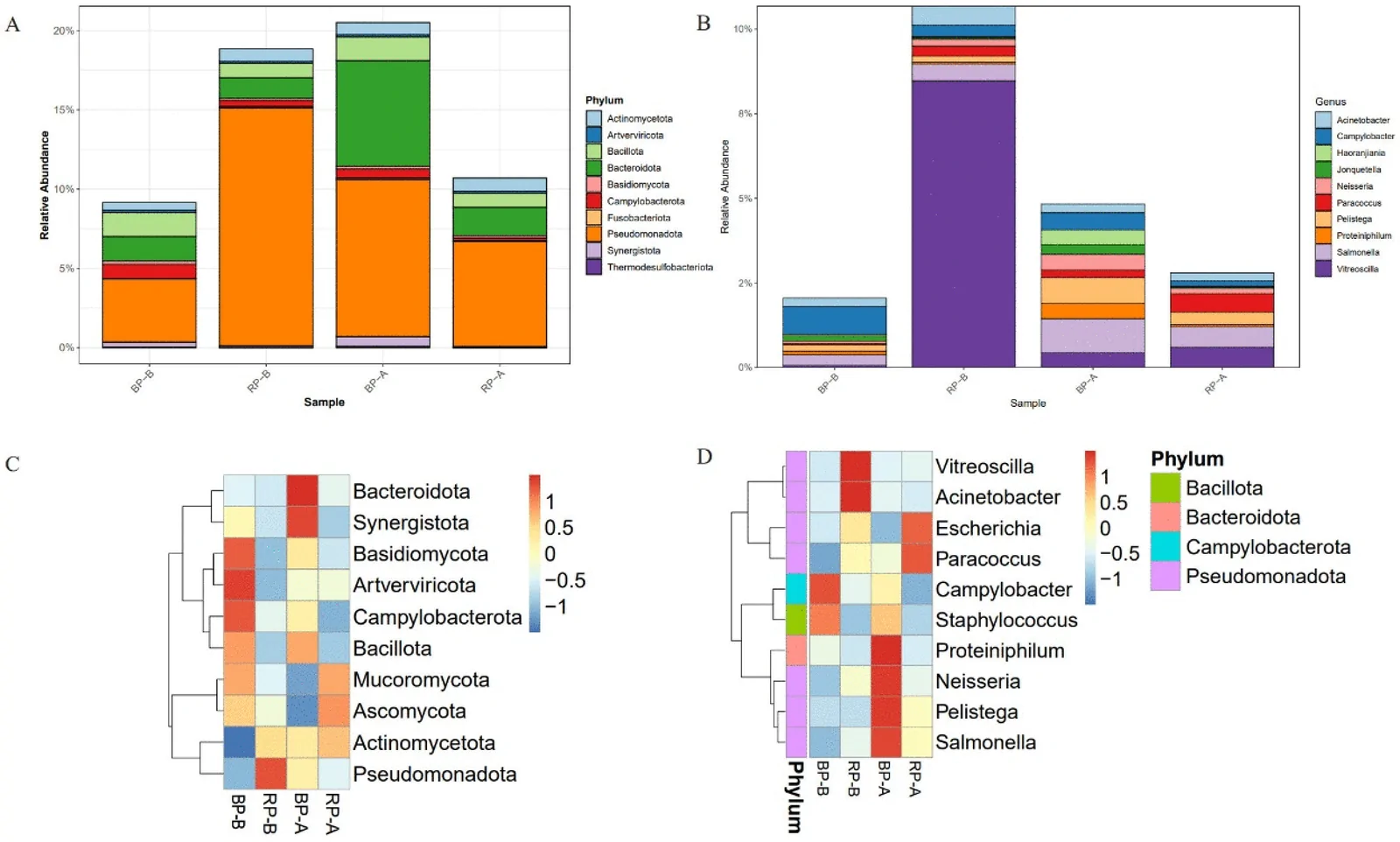
Gut microbiota composition in two python species. (A) Relative abundance bar plot at the phylum level. (B) Relative abundance bar plot at the genus level. (C) Heatmap showing relative abundance at the phylum level. (D) Heatmap showing relative abundance at the genus level.

At the genus level, the top five genera were *Vitreoscilla* (2.39% ± 2.03%), *Salmonella* (0.61% ± 0.15%), *Campylobacter* (0.46% ± 0.14%), *Pelistega* (0.38% ± 0.14%), and *Acinetobacter* (0.32% ± 0.08%) (Fig 3B, S4 Table).

Heatmap analysis showed that, at the phylum level, Bacteroidota, Synergistota, Actinomycetota, and Pseudomonadota exhibited increased relative abundance in BP-A compared to BP-B, while Pseudomonadota showed decreased abundance in RP-A compared to RP-B (Fig 3C).

At the genus level, *Proteiniphilum, Neisseria, Pelistega,* and *Salmonella* showed increased abundance in BP-A compared to BP-B, whereas *Vitreoscilla* and *Acinetobacter* exhibited decreased abundance in RP-A compared to RP-B (Fig 3D).

LEfSe analysis identified 51 differentially abundant taxa in BP-A, with taxa mainly belonging to Chitinophagia and *Salmonella*, whereas only 9 taxa were detected in BP-B (Fig 4A). In reticulated pythons, 6 differentially abundant taxa were identified in RP-B, compared to only 2 in RP-A, primarily belonging to *Yimella* (Fig 4B). Overall, BP-A exhibited a higher number of differentially abundant taxa than BP-B, whereas RP-A showed fewer taxa compared to RP-B.

**Fig 4:**
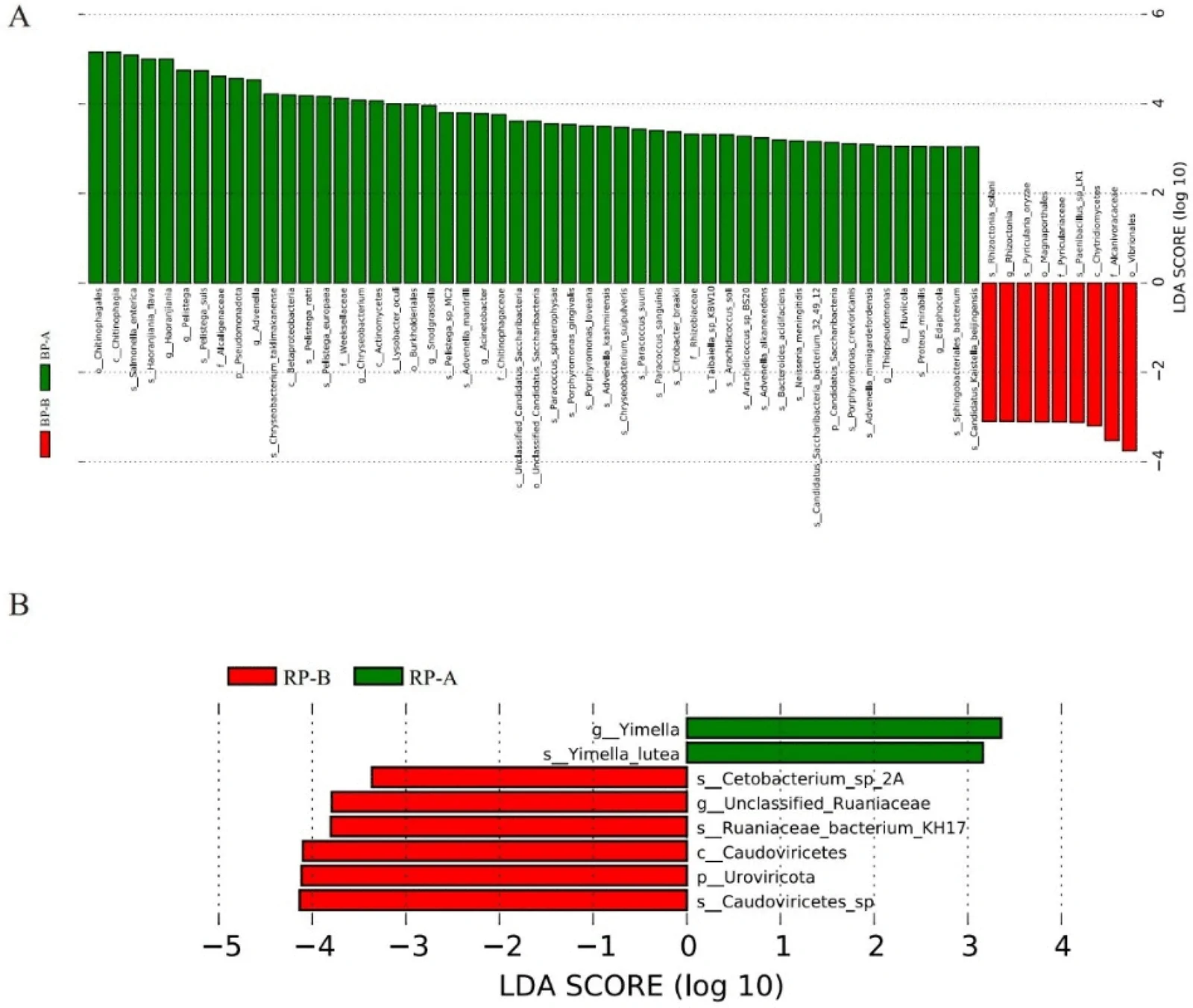
LEfSe analysis of gut microbiota in two python species. (A) Differentially abundant taxa between BP-B and BP-A in Burmese pythons (LDA score > 3). (B) Differentially abundant taxa between RP-B and RP-A in reticulated pythons (LDA score > 3). BP-B, Burmese pythons before enrichment; BP-A, Burmese pythons after enrichment; RP-B, reticulated pythons before enrichment; RP-A, reticulated pythons after enrichment.

### Functional analysis of gut microbiota

At KEGG level 2, functional differences were primarily associated with the category of Metabolism. Compared to BP-B, BP-A showed increased pathway abundance across multiple functional categories. In particular, pathways related to carbohydrate metabolism, glycan biosynthesis and metabolism, and nucleotide metabolism within the Metabolism category, as well as membrane transport within Environmental Information Processing, were more abundant. In contrast, compared to RP-B, most pathways exhibited decreased abundance in RP-A (Fig 5A, S5 Table).

**Fig 5:**
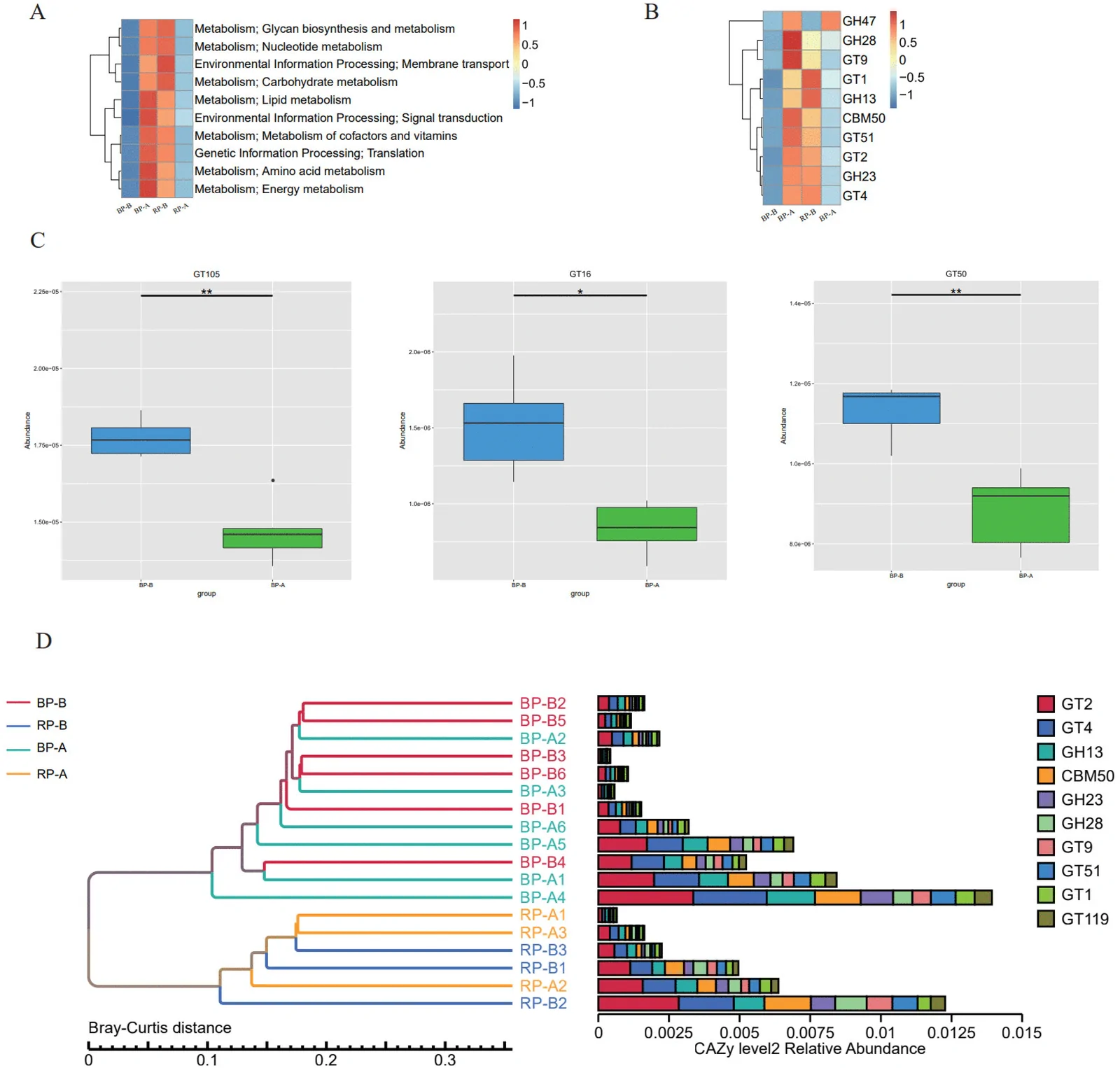
Functional profiles of gut microbiota. (A) Heatmap showing functional profiles at KEGG level 1 and level 2. (B) Heatmap showing CAZy family-level profiles. (C) Metastat analysis of CAZy families (* *p* < 0.05; ** *p* < 0.01). (D) Cluster analysis of samples based on CAZy profiles.

At the CAZy family level, glycoside hydrolases (GH) and glycosyltransferases (GT) showed notable variation between groups. Compared to BP-B, the abundance of GT1 and GH13 increased in BP-A. In contrast, compared to RP-B, GH47 showed no significant change, whereas most enzyme families exhibited decreased abundance in RP-A (Fig 5B, S6 Table). Metastat analysis identified three enzyme families that were significantly different between BP-B and BP-A. GT105 and GT50 showed highly significant differences, while GT16 showed a significant difference (Fig 5C). Cluster analysis indicated that samples from the same species tended to cluster together, whereas those from different species were more dispersed. GT2 and GT4 were the most abundant enzyme families across all samples. Some samples (e.g., BP-A4, BP-A1, and RP-B2) exhibited relatively higher overall abundances than others. Overall, the total abundance of CAZy enzyme families was higher in BP-A than in BP-B, but lower in RP-A compared to RP-B (Fig 5D).

### Antibiotic resistance genes

The top 10 most abundant antibiotic resistance genes (ARGs) were identified across all samples. Among them, *vanT_G* showed the highest relative abundance, followed by *Pul-EF-Tu*. Higher ARG abundance was observed in some samples (e.g., BP-A1 and BP-A4) compared to others. The detected ARG classes mainly included resistance to glycopeptides, quaternary ammonium compounds, protein synthesis inhibitors, β-lactams, tetracyclines, and sulfonamides. Glycopeptide and β-lactam resistance genes were more prominent in BP groups, whereas tetracycline and quaternary ammonium resistance genes were more represented in RP groups (Fig 6A).

**Fig 6:**
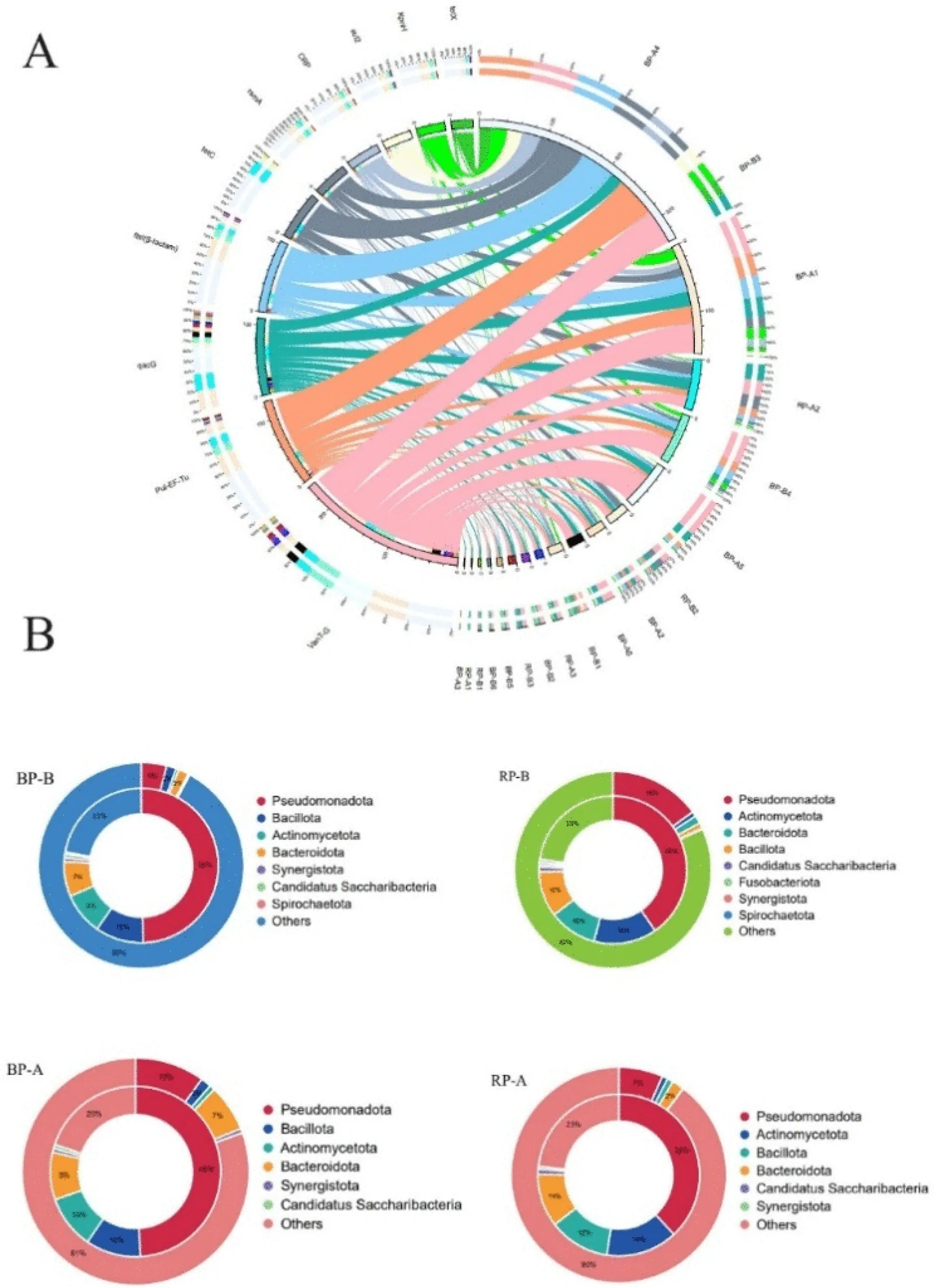
Distribution of antibiotic resistance genes (ARGs) in two python species. (A) Circos plot showing the distribution of ARGs across samples. The left panel represents ARG categories, and the right panel represents sample distribution. (B) Donut charts showing the taxonomic assignment of ARGs.

Taxonomic assignment analysis showed that ARGs were primarily associated with Pseudomonadota, Bacteroidota, and Bacillota across groups. In BP-B and BP-A, ARGs were mainly linked to Pseudomonadota, with additional contributions from Bacteroidota and Bacillota. In RP-B and RP-A, ARGs were predominantly associated with Pseudomonadota (Fig 6B).

## Discussion

In this study, differences in the responses to environmental enrichment were observed between *Python bivittatus* and *Malayopython reticulatus*. Environmental enrichment was associated with changes in the gut microbiome and resistome of captive pythons. The gut microbiota plays a critical role in host physiological regulation [23]. Together, these findings suggest that captive husbandry conditions may influence the composition of reptile-associated microbial communities, while host identity remains a key determinant of microbiome assembly [24–26]. This supports the view that environmental enrichment, through changes in diet and habitat complexity, can act as an ecological driver of microbiome restructuring, similar to patterns observed in other captive mammals [27].

In Burmese pythons, α-diversity, as indicated by the ACE and observed species indices, increased significantly after enrichment. In contrast, reticulated pythons exhibited a different pattern of community change. Such host-dependent responses are consistent with the concept that gut microbiota is shaped by both intrinsic host traits and extrinsic environmental factors [23,28]. Previous studies in reptiles have shown that host species and ecological habits can strongly influence gut microbial composition [29,30]. Similar patterns have also been reported across diverse reptilian taxa under comparable living conditions [31]. In snakes, host phylogeny and ecological traits may contribute to the establishment of species-specific microbial niches [32]. Therefore, the divergent responses observed here likely reflect the combined effects of host selection and enrichment-driven environmental change, rather than a uniform response to captive improvement.

At the taxonomic level, Pseudomonadota and Bacteroidota were the dominant phyla in both species, which is consistent with previous reptile microbiome studies [29,30]. Pseudomonadota comprises metabolically versatile taxa and has been associated with host adaptation and environmental responsiveness [33]. However, its overrepresentation may also indicate microbial imbalance under certain conditions [34]. Bacteroidota are well known for their roles in polysaccharide degradation and utilization of complex substrates [35,36]. They also play an important role in host–microbe interactions [37]. In our study, the relative abundance of Bacteroidota was higher after enrichment in Burmese pythons, which may reflect altered substrate availability and changes in the intestinal ecological niche under improved husbandry conditions. Evidence from human studies indicates that diet can influence gut microbial composition [38,39], while research in larval amphibians has shown that dietary tannins can alter the gut microbiome [40]. These findings provide general context for considering dietary influences on gut microbial communities. In the present study, the enrichment program included both a change in prey type and an increase in food quantity. These modifications may have altered the availability and composition of substrates for gut microorganisms, potentially contributing to the observed community changes, including the increase in the relative abundance of Bacteroidota in Burmese pythons. Dietary modification may therefore represent one of several contributing factors within the combined enrichment intervention.

Functional analysis showed that the enrichment may influence metabolic potential, particularly in pathways related to carbohydrate metabolism and environmental information processing. This is consistent with the view that microbiome function is highly responsive to changes in nutritional substrates and host environment [41]. In Burmese pythons, the increased abundance of glycoside hydrolases (GH) and glycosyltransferases (GT) suggests enhanced capacity for complex carbohydrate degradation and glycan processing within the gut ecosystem. Glycoside hydrolases are essential for polysaccharide breakdown and fermentation [35]. In contrast, glycosyltransferases are involved in extracellular polysaccharide synthesis and host–microbiome interface regulation [42]. The enrichment of GH and GT families may therefore suggest an increased microbial functional potential for substrate degradation and carbohydrate-related metabolism under enriched husbandry conditions. In addition, the increased abundance of membrane transport-related functions may indicate an increased potential for nutrient uptake and metabolite transport, potentially reflecting microbial responses to changes in nutrient availability [43].

In addition to compositional and functional changes, environmental enrichment was also associated with differences in the gut resistome. We observed that Pseudomonadota contributed the largest proportion of antibiotic resistance genes, which is consistent with its role as an important reservoir of resistance determinants and a major host of multidrug resistance genes [44–46]. The occurrence and dissemination of antibiotic resistance genes in captive environments have been associated with multiple factors, including diet and animal contact [47,48]. Environmental management practices, such as disinfectant exposure, have been proposed as potential factors influencing resistome dynamics through co-selection and enhanced horizontal gene transfer [49,50]. In particular, quaternary ammonium compounds can select for resistance-associated genes such as *qac* determinants and may promote co-selection with antibiotic resistance elements located on mobile genetic elements [51,52]. Therefore, the changes observed in the resistome of both python species may reflect the combined influence of altered feeding conditions, environmental exposure, and routine husbandry practices. The observed changes in the resistome of both python species may represent the combined effects of altered feeding conditions, environmental changes, and other husbandry-related factors associated with the enrichment program. It is worth noting that in this study, routine disinfection of the python enclosures was performed. No further statistical analysis was conducted regarding the type or dosage of the disinfectant. It should also be noted that antibiotics were used for veterinary treatment of some snakes during the study period. However, complete information regarding the specific antibiotics, dosage, treatment duration, and timing of administration was unavailable. Therefore, antibiotic exposure represents a potential confounding factor in the interpretation of the observed resistome patterns. Together with the incomplete information on disinfectant use, this limitation prevents us from determining whether the observed changes in antibiotic resistance genes were attributable specifically to environmental enrichment. Accordingly, the resistome findings should be interpreted cautiously.

Our study has several limitations. One important limitation is that the sample size for reticulated pythons was small, which may limit the generalizability of the findings. Another limitation is the absence of a concurrent un-enriched control group. Although sampling was conducted in April–May and October–November, the potential influence of temporal and seasonal factors cannot be completely excluded. Because the enrichment intervention coincided with this sampling interval, temporal or seasonal effects may have contributed to the observed changes. Future studies should include larger sample sizes, concurrent control groups, and stricter control of environmental temperature to strengthen causal inference. Furthermore, an additional limitation of this study is that the interval between the last feeding event and fecal sample collection was not systematically standardized across individuals. Differences in feeding status or the postprandial state at the time of sampling may have contributed to some of the observed microbial variation. Future studies should standardize and record the interval between feeding and fecal sampling to minimize this potential source of confounding. Besides, ARG interpretation based solely on metagenomic annotation requires further validation. Analyses of mobile genetic elements are important for assessing the potential for horizontal gene transfer [53,54]. In addition, metagenomic analyses of mobile genetic elements can provide insights into transmission potential and ecological risk [45]. Finally, because environmental enrichment was implemented as a combined intervention involving multiple simultaneous modifications, the contribution of individual enrichment components could not be evaluated independently. In addition, enrichment elements such as soil, vegetation, and flowing water may have directly introduced environmental microorganisms into the enclosure, potentially contributing to the observed microbial shifts. Future studies should incorporate larger sample sizes, concurrent control groups, multiple independent enclosures, stricter environmental control, and longitudinal multi-omics approaches to better distinguish enrichment-associated effects from temporal and environmental variation.

## Conclusion

This study suggests that environmental enrichment may influence the gut microbiome and resistome of captive pythons in a host-dependent manner. By applying shotgun metagenomic sequencing, we showed that enrichment was associated with changes in microbial diversity, taxonomic composition, and functional potential, particularly in pathways related to carbohydrate metabolism and environmental information processing. In addition, enrichment was associated with differences in the gut resistome, with Pseudomonadota contributing the majority of antibiotic resistance genes. These findings suggest that improvements in captive environments might not only influence animal behavior and welfare but also extend to the microbial ecosystems associated with the host.

## Supporting information

S1 Table Sampling information. (XLSX)

S2 Table Metagenomic sequencing information. (DOCX)

S3 Table α-diversity analysis. (XLSX)

S4 Table Relative abundance of communities at the phylum and genus levels. A. Phylum level. B. Genus level.
(DOCX)

S5 Table Relative abundance at KEGG level 2. (XLSX)

S6 Table Relative abundance at CAZy family level. (XLSX)
