# Supplementary material for: Environmental enrichment is associated with changes in the gut microbiome and resistome of captive pythons
Source: PLoS One. 2026 Nov 4;21(11):e0360735. doi: 10.1371/journal.pone.0360735 (PMC13734490; doi:10.1371/journal.pone.0360735)
Supplement: Supplementary file 1 [file pone.0360735.s002.docx]

| SampleID | Raw_Base(G) | Clean_Base(G) | Clean_Q20(%) | Clean_Q30(%) | Clean_GC(%) | Effective(%) |
| --- | --- | --- | --- | --- | --- | --- |
| BP-B1 | 7.51 | 7.42 | 97.69 | 93.34 | 41.19 | 98.76 |
| BP-B2 | 6.28 | 6.18 | 97.57 | 92.92 | 41.08 | 98.45 |
| BP-B3 | 7.41 | 7.31 | 97.72 | 93.4 | 41.06 | 98.65 |
| BP-B4 | 6.82 | 6.74 | 97.67 | 93.12 | 41.28 | 98.85 |
| BP-B5 | 7.07 | 6.96 | 97.62 | 93.01 | 41.24 | 98.44 |
| BP-B6 | 7.31 | 7.22 | 97.64 | 93.11 | 40.83 | 98.79 |
| RP-B1 | 7.22 | 7.15 | 97.67 | 93.18 | 42.42 | 99.08 |
| RP-B2 | 6.88 | 6.8 | 97.89 | 93.76 | 44.21 | 98.82 |
| RP-B3 | 7.64 | 7.56 | 97.78 | 93.52 | 41.62 | 98.92 |
| BP-A1 | 6.98 | 6.9 | 97.79 | 93.46 | 42.81 | 98.86 |
| BP-A2 | 6.76 | 6.68 | 97.62 | 93.07 | 40.6 | 98.86 |
| BP-A3 | 7.15 | 7.03 | 97.6 | 92.87 | 42.72 | 98.36 |
| BP-A4 | 7.09 | 6.99 | 97.72 | 93.41 | 42.31 | 98.63 |
| BP-A5 | 6.98 | 6.88 | 97.52 | 92.72 | 41.3 | 98.64 |
| BP-A6 | 8.08 | 8.02 | 97.84 | 93.6 | 45.45 | 99.28 |
| RP-A1 | 7.88 | 7.81 | 97.65 | 93.07 | 41.28 | 99.13 |
| RP-A2 | 7 | 6.93 | 97.74 | 93.39 | 43.13 | 98.92 |
| RP-A3 | 7.38 | 7.31 | 97.71 | 93.3 | 41.08 | 99.06 |

S2 Table metagenomics sequencing information
