# Supplementary material for: Environmental enrichment is associated with changes in the gut microbiome and resistome of captive pythons
Source: PLoS One. 2026 Nov 4;21(11):e0360735. doi: 10.1371/journal.pone.0360735 (PMC13734490; doi:10.1371/journal.pone.0360735)
Supplement: Supplementary file 2 [file pone.0360735.s004.docx]

| phylum | BP-B | RP-B | BP-A | RP-A |
| --- | --- | --- | --- | --- |
| p__Pseudomonadota | 0.03996 | 0.150142 | 0.099037 | 0.066281 |
| p__Bacteroidota | 0.015513 | 0.013169 | 0.066657 | 0.017892 |
| p__Campylobacterota | 0.008958 | 0.003827 | 0.005733 | 0.001852 |
| p__Bacillota | 0.01522 | 0.008911 | 0.015007 | 0.008848 |
| p__Synergistota | 0.003016 | 0.001085 | 0.006218 | 0.00058 |
| p__Actinomycetota | 0.004654 | 0.008009 | 0.007687 | 0.008289 |
| p__Fusobacteriota | 0.000132 | 0.000745 | 0.000886 | 0.000411 |
| p__Basidiomycota | 0.002106 | 0.001293 | 0.00177 | 0.00142 |
| p__Thermodesulfobacteriota | 0.000414 | 0.000046 | 0.000747 | 0.000106 |
| p__Artverviricota | 0.001573 | 0.001194 | 0.001329 | 0.001316 |
| Others | 0.908454 | 0.811579 | 0.794929 | 0.893005 |
| genus | BP-B | RP-B | BP-A | RP-A |
| *g__Vitreoscilla* | 0.000617 | 0.084711 | 0.004363 | 0.005921 |
| *g__Campylobacter* | 0.008323 | 0.003451 | 0.005142 | 0.001548 |
| *g__Salmonella* | 0.003176 | 0.004996 | 0.01007 | 0.006186 |
| *g__Pelistega* | 0.001904 | 0.001854 | 0.007679 | 0.003727 |
| *g__Paracoccus* | 0.000361 | 0.002952 | 0.002249 | 0.005428 |
| *g__Haoranjiania* | 0.000514 | 0.000221 | 0.004319 | 0.000312 |
| *g__Neisseria* | 0.000765 | 0.002071 | 0.004619 | 0.001636 |
| *g__Acinetobacter* | 0.002449 | 0.005587 | 0.002571 | 0.00239 |
| *g__Proteiniphilum* | 0.000983 | 0.000465 | 0.004443 | 0.000547 |
| *g__Jonquetella* | 0.001399 | 0.000496 | 0.002859 | 0.000267 |
| Others | 0.979509 | 0.893196 | 0.951686 | 0.972038 |

S4 Table Relative abundance of communities at the phylum and genus levels
